# Insulin‐Like Growth Factor 2 mRNA Binding Protein 2 Promotes HBV‐Associated Hepatocellular Carcinoma Progression by Enhancing Heme Oxygenase 1 Stability in an M^6^A‐dependent Manner

**DOI:** 10.1002/mco2.70371

**Published:** 2025-08-31

**Authors:** Yan Zhao, Yan Cui, Hongxiu Qiao, Sandra Chiu, Xia Chuai

**Affiliations:** ^1^ Department of Pathogenic Biology Hebei Medical University Shijiazhuang China; ^2^ State Key Laboratory of Virology and Biosafety, Wuhan Institute of Virology, Center for Biosafety Mega Science Chinese Academy of Sciences Wuhan China

1

Dear Editor,

Chronic hepatitis B virus (HBV) infection has been established as a primary etiological factor in hepatocarcinogenesis. N6‐methyladenosine (m^6^A), the predominant modification of eukaryotic RNAs, has been shown to play a critical role in both HBV life cycle and HBV‐associated hepatocarcinogenesis [1]. This reversible modification exerts its biological effects through specialized RNA‐binding proteins (“readers”) that specifically recognize m^6^A motifs and regulate RNA metabolism processes [2]. As a newly identified m^6^A reader, insulin‐like growth factor 2 mRNA binding protein 2 (IGF2BP2) has been shown to promote tumorigenesis by enhancing the stability of its target transcripts [3]. Despite these findings, the role of IGF2BP2 in the specific pathogenesis of HBV‐associated hepatocellular carcinoma (HCC) remains poorly understood. Therefore, our study aimed to systematically investigate the function of IGF2BP2 in HBV‐associated hepatocarcinogenesis and evaluate its potential as a molecular target for HBV‐associated HCC intervention.

First, we analyzed the correlation between the expression of IGF2BP2 and HBV‐associated HCC (HBV‐HCC). We collected liver tissue samples from HCC patients at the Third Hospital of Hebei Medical University (Hebei Province, China). The results revealed that the expression of IGF2BP2 in the liver tissue samples from both HCC groups was significantly higher than that in the paired adjacent normal tissue samples (Figure 1A). Additionally, the expression of IGF2BP2 in liver samples from the HBV‐HCC group was also significantly higher than that in those from the HBV‐negative group (Figure 1A). Furthermore, we detected the expression of IGF2BP2 in HBV‐replicating HCC cells. The results demonstrated that the protein expression level of IGF2BP2 was also significantly elevated in HBV‐replicating cells (Figure 1A).

**FIGURE 1:**
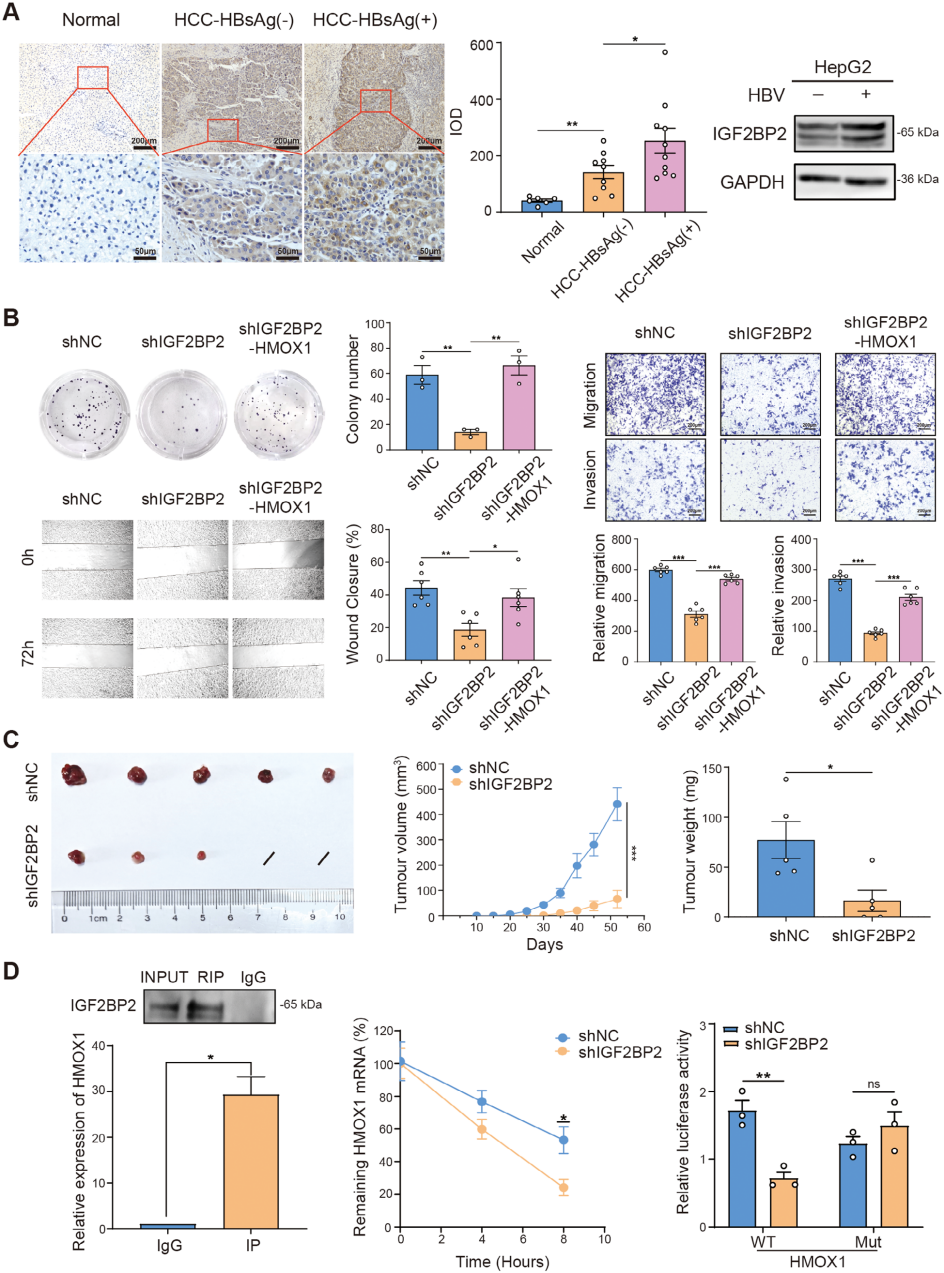
IGF2BP2 promotes HBV‐associated hepatocellular carcinoma (HCC) progression through m^6^A‐dependent stabilization of HMOX1. (A) Comparative analysis of IGF2BP2 expression levels (the integrated optical density [IOD] per field was calculated for quantification) in hepatocellular carcinoma (HCC) tissues and adjacent non‐tumor liver tissues from HCC patients (*n* = 6–9 per group), and western blot detection of IGF2BP2 expression in HBV‐replicating HCC cells (*n* = 3). (B) Functional characterization of IGF2BP2 knockdown in HepG2 cells through colony formation assay (*n* = 3), wound healing assay (*n* = 6), and transwell migration assay (*n* = 6), demonstrating that IGF2BP2 depletion significantly impairs proliferative, migratory, and invasive capacities. (C) In vivo tumorigenicity assessment showing reduced xenograft growth in nude mice subcutaneously transplanted with shIGF2BP2 HepG2 cells (*n* = 5 mice/group). Quantitative analysis revealed significant reductions in both tumor volume and weight. (D) RNA immunoprecipitation (RIP)‐qPCR, RNA stability assay, and dual‐luciferase reporter assays (*n* = 3) (WT: wild type of HMOX1 mRNA; Mut: a mutation of the predicted m^6^A site (A185C) on HMOX1 mRNA) were used to delineate the IGF2BP2‐HMOX1 interaction and validate m^6^A‐dependent post‐transcriptional regulation. *p < 0.05, **p < 0.01, ***p < 0.001.

Chronic HBV infection has been recognized as a major risk factor for HCC, and HBV‐associated HCC is more aggressive than HCC caused by other factors [4]. To further clarify whether IGF2BP2 is involved in regulating the progression of HBV‐HCC, we used a pCS‐HBV1.3 plasmid‐transfected HepG2 model to evaluate the effect of IGF2BP2 on the biological behavior of HBV‐HCC. The results revealed that downregulating IGF2BP2 using shRNA significantly inhibited the proliferation, migration, and invasion capabilities of HBV‐replicating HepG2 cells (Figure 1B). To further explore the role of IGF2BP2 in HCC progression in vivo, we performed xenograft tumor experiments by subcutaneously injecting shNC‐ or shIGF2BP2‐transfected cells into nude mice (datails in Supplementary Information). We found that IGF2BP2 depletion significantly inhibited HCC growth, as reflected by reduced tumor volume and tumor weight (Figure 1C). In summary, these data demonstrate that IGF2BP2 promotes HBV‐HCC progression.

Since sorafenib and apatinib are both widely used to treat HCC, the issue of drug resistance in HCC cells during clinical treatment has garnered increasing attention. We further examined whether changes in IGF2BP2 expression affect the sensitivity of HCC cells to these two drugs. We treated shIGF2BP2‐transfected HepG2 cells with sorafenib or apatinib at their IC_50_ concentrations and found that IGF2BP2 knockdown enhanced the inhibitory effects of sorafenib and apatinib on HCC cells.

To further explore the mechanism by which IGF2BP2 promotes HBV‐HCC tumorigenicity, we used bioinformatics analysis (https://starbase.sysu.edu.cn) to screen several downstream genes associated with HCC that can bind to IGF2BP2. Additionally, since the above results have shown that the overexpression of IGF2BP2 enhances the resistance of HepG2 cells to sorafenib and apatinib, and it was confirmed that the antitumor effects of sorafenib and apatinib are related to ferroptosis [5]. We focused on genes related to ferroptosis. Using qPCR, we found that the expression of heme oxygenase 1 (HMOX1), a key molecule involved in ferroptosis, was most significantly reduced (more than twofold change) in shIGF2BP2‐transfected HepG2 cells. We further overexpressed HMOX1 in shIGF2BP2‐transfected HepG2 cells and found that the inhibitory effect of IGF2BP2 downregulation on the proliferation, migration, and invasion of HCC cells was reversed (Figure 1B).

To elucidate IGF2BP2‐HMOX1 interaction, RIP‐qPCR was performed, demonstrating the direct binding of IGF2BP2 to HMOX1 mRNA (Figure 1D). Moreover, the shortened HMOX1 half‐life was investigated following IGF2BP2 knockdown, confirming the role of IGF2BP2 in stabilizing HMOX1 mRNA (Figure 1D). To further determine the mechanism of IGF2BP2‐HMOX1 interaction, MeRIP‐qPCR was used to verify m^6^A modification in HMOX1 mRNA (details in Supplementary Information). Bioinformatic analysis using SRAMP predicted A185 as a high‐confidence m^6^A site on HMOX1 mRNA. Subsequently, a mutation was generated at the predicted m^6^A site (A185C). Using a luciferase reporter assay, it was found that luciferase activity was significantly attenuated in HMOX1‐WT cells upon IGF2BP2 knockdown, whereas in HMOX1‐mut cells, it was not affected (Figure 1D). These findings demonstrate that IGF2BP2 promotes HBV‐HCC progression in an m^6^A‐dependent manner by stabilizing HMOX1 mRNA.

In summary, our study reveals for the first time that IGF2BP2 is involved in the development of HBV‐HCC. Our findings provide a new perspective on HBV‐HCC development. IGF2BP2 might be a potential therapeutic target for HBV‐HCC. Currently, this study has confirmed that IGF2BP2 facilitates the progression of HBV‐HCC by targeting HMOX1 in an m^6^A‐dependent manner. However, whether IGF2BP2 promotes HBV replication and how its interaction with HMOX1 affects the development of HBV‐HCC remain to be further studied.

## Author Contributions

**Xia Chuai**: conceptualization and supervision. **Sandra Chiu**: conceptualization and supervision. **Yan Zhao**: data curation, formal analysis, investigation, methodology, visualization, writing – original draft. **Yan Cui**: data curation, formal analysis, investigation, methodology, validation. **Hongxiu Qiao**: data curation, investigation, methodology, visualization. **Yan Zhao**: writing, review, and editing. **X. Chuai**: writing, review, and editing. **S. Chiu**: writing, review, and editing. All the authors contributed to the manuscript and approved the submitted version.

## Ethics Statement

The study protocol was performed in accordance with the Declaration of Helsinki and approved by the Medical Ethics Committee of Hebei Medical University (approval number: 2021100). Written informed consent was obtained from all participants. All the animal research was carried out in accordance with the rules of Basel Declaration and approved by the Ethics Board of the Animal Ethics Committee of Hebei Medical University (approval number: IACUC‐Hebmu‐2023007).

## Conflicts of Interest

The authors declare no conflicts of interest.

## Supporting information




**Supporting information file 1**: mco270371‐sup‐0001‐SuppMat.docx

## Data Availability

All the data generated or analyzed during this study are included in this article and its Supporting Information. Further inquiries can be directed to the corresponding authors.
