# Supplementary material for: Insulin‐Like Growth Factor 2 mRNA Binding Protein 2 Promotes HBV‐Associated Hepatocellular Carcinoma Progression by Enhancing Heme Oxygenase 1 Stability in an M6A‐dependent Manner
Source: MedComm (2020). 2025 Aug 31;6(9):e70371. doi: 10.1002/mco2.70371 (PMC12399564; doi:10.1002/mco2.70371)
Supplement: Supplementary file 1 — Supporting information file 1: mco270371‐sup‐0001‐SuppMat.docx [file MCO2-6-e70371-s001.docx]

Supplementary Information for

**Insulin like Growth Factor 2 mRNA binding Protein 2 Promotes HBV-associated Hepatocellular Carcinoma Progression by Enhancing Heme Oxygenase 1 Stability in an m^6^A-dependent Manner**

Yan Zhao^1#^, Yan Cui^1#^, Hongxiu Qiao^1#^, Sandra Chiu^1^*, Xia Chuai^1, 2^*

1 Department of Pathogenic Biology, Hebei Medical University, Shijiazhuang 050017, China.

2 State Key Laboratory of Virology and Biosafety, Wuhan Institute of Virology, Center for Biosafety Mega Science, Chinese Academy of Sciences, Wuhan 430207, China.

# Yan Zhao, Yan Cui, and Hongxiu Qiao contributed equally to this work.

***Correspondence**

Xia Chuai, State Key Laboratory of Virology and Biosafety, Wuhan Institute of Virology, Center for Biosafety Mega Science, Chinese Academy of Sciences, Wuhan 430207, China. Email: chuaixia@wh.iov.cn

Sandra Chiu, Department of Pathogenic Biology, Hebei Medical University, Shijiazhuang 050017, China. qiux@ustc.edu.cn

**MATERIALS AND METHODS**

**Clinical specimen collection**

Liver tissue samples were obtained intraoperatively from HCC patients (pathologically confirmed, excluding multi-primary malignancies) under informed consent at the Third Hospital of Hebei Medical University (Shijiazhuang, China). Patients were stratified into HBsAg-negative (n = 9) and HBsAg-positive (n = 9) cohorts. Adjacent non-tumorous liver tissues from HBsAg-negative patients served as controls (n = 6). The study protocol was performed in accordance with the Declaration of Helsinki and approved by the Institutional Review Board of Hebei Medical University (Approval No. 2021100).

**Plasmid construction**

The plasmid pCS-HBV1.3 was constructed by inserting a 1.3-fold-overlength HBV genome (ayw subtype; GenBank: AB267090.1) into the *Pst*Ⅰ site of the pCS-CG vector. The IGF2BP2 and HMOX1 overexpression plasmid was constructed by inserting the IGF2BP2 or HMOX1 coding sequence into the *EcoR*I site of the pcDNA3.1 vector.

**Cell culture and transfection**

HepG2 and HEK293T cell lines were obtained from the National Collection of Authenticated Cell Cultures (Shanghai, China). HepG2 cells used in this study were maintained in MEM containing 10% FBS under standard culture conditions (37°C, 5% CO_2_). For transient transfection experiments, cells were seeded in 6-well plates and transfected at 80% confluency using Lipofectamine™ 2000 with either plasmid DNA or gene-specific siRNA (Target sequence of siIGF2BP2: CUCUCGGGUAAAGUGGAAU) following manufacturer's protocols.

**Stable cell line establishment**

Lentiviral vectors pCDH expressing IGF2BP2-targeting short hairpin RNA (shRNA) (shIGF2BP2: 5′-GGATGATGAACAAGCTTTA-3′) or non-targeting control sequences (shNC: 5′-GGTGAAGGTGATGCAACATAC-3′) were constructed. Viral particles were produced by co-transfecting packaging plasmids (pLP1, pLP2, pVSV-G) with pCDH constructs in HEK293T cells, followed by supernatant concentration. HepG2 cells were infected with pseudoviral particles and selected with 5 μg/mL puromycin. Stable knockdown efficiency was validated by qRT-PCR and Western blot prior to experimental applications.

**Immunohistochemistry**

Deparaffinized and rehydrated tissue sections underwent antigen retrieval and blocking (1 h, room temperature). Slides were incubated overnight at 4°C with primary antibodies: anti-IGF2BP2 and anti-Ki67 (Proteintech, USA). After PBS washes, secondary antibodies were applied. Protein expression was visualized using a Leica DMC4500 microscope (Leica, Germany) and semi-quantified via Image-Pro Plus 6.0. Integrated optical density (IOD) per field was calculated from five non-overlapping 400× fields per section.

**Quantitative Real-Time PCR (qRT-PCR)**

Total RNA was extracted from tissue/cell samples using TRIzol Universal Reagent (TianGen Biotech, Beijing, China) and reverse transcribed with PrimeScript RT Master Mix (Takara Bio, Japan). qPCR was performed on QuantStudio™ 3 System (Thermo Fisher, USA) using SYBR Premix Ex Taq™ (Takara) with gene-specific primers: GAPDH: F: GTGGACCTGACCTGCCGTCTAG, R: GAGTGGGTGTCGCTGTTGAAGTC; IGF2BP2: F: AGTGGAATTGCATGGGAAAATCA, R: CAACGGCGGTTTCTGTGTC; HMOX1: F: CCCAGGCAGAGAATGCTGAG, R: ATGTGGTACAGGGAGGCCAT.

**Colony formation assay**

Cells (2×10³/well) from each group were plated into 6-well plates and cultured for 10 days at 37°C with 5% CO₂. Colonies were fixed, stained with 0.1% crystal violet, and quantified by counting visible colonies (>40 µm diameter) from captured images.

**Scratch wound assay**

Cells seeded in 6-well plates were scratched with a sterile pipette tip 48 h post-transfection. Reference marks ensured consistent imaging positions. Cells were maintained in serum-free medium containing 1 mM mitomycin to suppress proliferation. Migration into the wound area was assessed by comparing phase-contrast images taken at 0 h and 72 h post-scratch, with migration rates calculated based on wound closure.

**Migration and invasion assays**

For migration: Cells (1×10^5^) in serum-free MEM were seeded into Transwell® chambers (8 μm pores; Corning). For invasion: Matrigel-coated chambers (BD BioCoat™) were used. Both assays employed 20% FBS-containing medium as chemoattractant in the lower chamber. After 24 h, transmigrated cells on membrane undersides were fixed, crystal violet-stained, and counted microscopically.

**Mouse xenograft tumor model**

Four-week-old male BALB/c nude mice were purchased from Beijing HFK Bioscience Co., Ltd., and were maintained under specific pathogen-free conditions. HepG2 with stable IGF2BP2 knockdown (HepG2-shNC as control) were harvested and suspended in MEM, and 2×10⁶ cells in 100 μL of PBS were subcutaneously injected into the armpit of nude mice (n=5 each group). Tumor volume was monitored every 5 days using the formula: Volume (mm³) = (length × width²)/2. Mice were euthanized at day 52 for tumor excision, weighing, and downstream analyses. All procedures were approved by Hebei Medical University's Animal Ethics Committee (IACUC protocol number: 2023007).

**RNA stability assay**

Cells were seeded in 6-well plates to reach 50% confluency 24 h post-transfection. Actinomycin D (5 μg/ml) was added, and cells were harvested at designated time points. Total RNA was extracted and analyzed by RT-PCR to estimate mRNA turnover rates and half-lives.

**RNA immunoprecipitation (RIP)**

RIP was performed using an RNA immunoprecipitation kit (Geneseed, China) following the manufacturer’s protocol. Anti-IGF2BP2 antibody (5 μg, Proteintech) or control IgG was conjugated to protein A/G magnetic beads (Thermo Fisher) via 4-hour incubation at 4°C and three washes. Precleared lysates in RIP buffer were incubated with antibody-bound beads. Coimmunoprecipitated RNAs were quantified by RT-qPCR.

**Methylated RNA immunoprecipitation and qPCR (MeRIP-qPCR)**

Total RNA was fragmented (~100 nt) and immunoprecipitated with an m^6^A antibody using the EpiQuik™ CUT&RUN m^6^A RNA Enrichment Kit (Epigen Tek). Enriched m^6^A-containing mRNAs were quantified by RT-qPCR.

**Statistical analysis**

Data represent at least three independent experiments performed in triplicate (unless specified), expressed as mean ± SEM. Statistical significance (*P*< 0.05) was determined by Student’s t-test (GraphPad Prism 8.0, MA, USA).
